# Research on vehicle scheduling for forest fires in the northern Greater Khingan Mountains

**DOI:** 10.1038/s41598-025-85638-3

**Published:** 2025-01-11

**Authors:** Jie Zhang, Junnan He, Shihao Ren, Pei Zhou, Jun Guo, Mingyue Song

**Affiliations:** 1 College of Energy and Transportation Engineering, Inner Mongolia Agricultural University, Hohhot, 010010 China; 2 School of Transportation and Logistics Engineering, Wuhan University of Technology, Wuhan, 430070 China; 3 College of Forestry, Inner Mongolia Agricultural University, Hohhot, 010010 China

**Keywords:** Emergency relief, Improved genetic algorithms, Vehicle scheduling, Chaos Search, Computer science, Computational science

## Abstract

In the face of forest fire emergencies, fast and efficient dispatching of rescue vehicles is an important means of mitigating the damage caused by forest fires, and is an effective method of avoiding secondary damage caused by forest fires, minimizing the damage caused by forest fires to the ecosystem, and mitigating the losses caused by economic development. this paper takes the actual problem as the starting point, constructs a reasonable mathematical model of the problem, for the special characteristics of the emergency rescue vehicle scheduling problem of forest fires, taking into account the actual road conditions in the northern pristine forest area, through the analysis of the cost of paths between the forest area and the highway, to obtain the least obstructed rescue paths, to narrow the gap between the theoretical model and the problem of the actual. Improvement of ordinary genetic algorithm, design of double population strategy selection operation, the introduction of chaotic search initialization population, to improve the algorithm’s solution efficiency and accuracy, through the northern pristine forest area of Daxing’anling real forest fire cases and generation of large-scale random fire point simulation experimental test to verify the effectiveness of the algorithm, to ensure that the effectiveness and reasonableness of the solution to the problem of forest fire emergency rescue vehicle scheduling program. It enriches the solution method of forest fire emergency rescue vehicle dispatching problem in Great Khingan area, which is of great significance to improve the emergency rescue capability in case of sudden forest fire. Through simulation experiments, the proposed Improved Genetic Algorithm (IGA) achieved an average rescue time reduction of 8.5% compared to conventional Genetic Algorithm (GA) and 3.5% compared to Improved Artificial Bee Colony (IABC) algorithm, with an average solution time of 9.4 ms.

## Introduction

In the context of global warming, the damage caused by forest fires is increasing, significantly impacting the global ecological environment and socio-economic systems^1^. Rising temperatures, intensified droughts, and irregular precipitation patterns, among other climate factors, make forests more flammable, creating favorable conditions for forest fires. Forests in various regions around the world have been affected by forest fires to varying degrees, causing severe impacts on ecosystems, air quality, and climate change.

The Greater Khingan Mountains region, as an important ecological protection area in northern China, is also one of the regions at high risk of forest fires in the country, deeply threatened by forest fires. Assessing fire hazards is essential for developing effective fire prevention strategies, as emphasized by Sakellariou et al.^2^, particularly in regions with vulnerable ecosystems. Furthermore, resilience-building frameworks proposed by Tampekis et al. offer a critical pathway to adapt to and mitigate wildfire risks under climate change. The unique geographical location and climatic conditions of the Greater Khingan Mountains make this area particularly susceptible to the effects of climate change, such as alternating extreme low and high temperatures. Additionally, the influence of the monsoon climate makes the vegetation more vulnerable to drought. Furthermore, the rich forest resources in the Greater Khingan Mountains attract significant human activity, but unregulated development and land use have increased the probability of fires occurring.

In the process of rescuing forest fires, fire trucks play a crucial role. The variability of terrain, uncertainty of fire sources, and urgency of rescue operations make the efficient and rapid scheduling of fire trucks a highly complex and pressing research challenge. Scientifically and reasonably planning the dispatch of fire trucks can not only shorten the response time, minimize losses, but also improve overall rescue efficiency, ensuring that the fire is controlled promptly and effectively. Building on previous studies, this work proposes a novel Improved Genetic Algorithm (IGA) that leverages chaotic search and power function carriers to optimize vehicle dispatch in forest fire scenarios. By integrating principles of functional complex systems science, this study explores innovative approaches to enhance fire management strategies and resilience-building frameworks.

## Methods

### Overview

Currently, there are numerous studies on the dispatch of emergency rescue vehicles after disasters. For instance, Zhang Zongjia et al.^3^ studied the emergency dispatch problem under flood disasters. Yan Sen et al.^4^ considered the situation of damaged roads and established a bi-objective model minimizing total cost and total time, which effectively improved the efficiency of emergency logistics and reduced losses caused by earthquakes. Wang Fuyu et al.^5^ took into account the fuzzy nature of emergency supplies demand at disaster points and constructed a multi-objective fuzzy optimization model aimed at minimizing the losses of affected people, minimizing the variance in satisfaction of emergency supplies at disaster points, and minimizing emergency rescue costs. Luo Hongsen et al.^6^ considered the urgency of different waterlogging points, path distances, and operation times to establish a multi-objective optimization model that effectively improved scheduling efficiency and reduced waterlogging losses.

In Western academia, research in the field of emergency rescue and vehicle dispatch started early, with scholars and research institutions actively exploring advanced technologies and methods to improve rescue efficiency and reduce losses. Payakorn Saksuriya et al.^7^ proposed a Vehicle Routing Problem with Time Windows (VRPTW) for the home healthcare routing problem, with compatibility matching constraints and total completion time as the objective functions. Fahmy Sherif et al.^8^ addressed the Split-Delivery Vehicle Routing Problem (SDVRP) in a typical supply chain for the collection, processing, and distribution of goods at aggregation centers. Armando Teles Dauer et al.^9^ proposed a Multi-Depot Vehicle Routing Problem (MDVRP) considering heterogeneous fleets and time windows.

In the study of scheduling rescue vehicles for forest fires, scholars such as Yang Zhongzhen et al.^10^ have researched the scheduling of fire trucks for forest fires, using a clonal immune algorithm to establish a multi-objective optimization model, thereby improving the emergency resource scheduling system in forest fire rescues. Wu Peng et al.^11^ introduced resource constraints into the emergency resource scheduling for forest fires and analyzed the rescue priorities of fire sites. Tian et al.^12^ improved the traditional ant colony algorithm by redesigning the heuristic information calculation method and the pheromone update function, introducing the concept of time windows to limit the completion time of urgent tasks, effectively solving the path planning model problem for forest fire logistics support. Zhang et al.^13^ based on the innovative concepts of normalization and superposition, integrated NDVI (Normalized Difference Vegetation Index) and slope weight factors, combined with satellite imagery and elevation data of the fire area to improve the A* path planning algorithm.

Building upon existing research, this study conducts cost-path analysis of terrain between highways and forested areas. By incorporating a chaotic search mechanism and power function carriers into an improved genetic algorithm, this study addresses the unique challenges of vehicle scheduling in forest fire rescue, offering a robust solution framework that enhances response efficiency in complex terrains. It aims to plan a path with minimal obstruction for rescue operations between forested areas and accessible roads. Subsequently, considering the specific characteristics of forest fire incidents, a problem model is constructed by integrating traditional Vehicle Routing Problem (VRP) formulations. An improved genetic algorithm based on power function carriers is designed to efficiently solve the emergency rescue vehicle scheduling problem for forest fires.

## Model parameter

The model parameters are shown in Table 1 below.

**Table 1:** To provide a clearer description of the problem, the following table lists the model parameters and their meanings that will be used in this paper.

$i,j$	Fire point number
$k$	Fire engine number
K = {1, 2……, $k$}	Fire trucks assembled
|K|	Number of fire engines
${d}_{i,}$	Distance between ignition points i and j
${v}_{k}$	Average speed of fire engines
${v}_{\text{f}}^{k}$	Extinguishing speed of fire engines
${v}_{i}$	Rate of fire spread at the point of ignition
${T}_{0}$	The moment of departure of the fire trucks
${T}_{k}$	Maximum travel time for fire engines
${F}_{\text{i}}$	Priority of rescue at ignition point i (Pi): [0, 1], determined by normalizing the fire spread rate of each fire point
$\text{M}$	A positive number
${x}_{\text{i},\text{j}}^{k}$	Decision variables, If firefighting vehicle k travels from node i to node j,then ${x}_{\text{i},\text{j}}^{k}$=1,otherwise 0
${t}_{\text{i}}^{k}$	Time for firefighting vehicle k to reach fire point i
${t}_{\text{i}}$	Time to extinguish a fire
${T}_{max}$	Total fire engine rescue time
${k}_{s}$	Correction factor for combustibles
${k}_{w}$	wind factor
${k}_{\varphi }$	Terrain slope factor
${v}_{0}$	Initial fire spread rate
$T$	Temperature at the fire point
${v}_{\text{w}}$	Air velocity

## Fire spread model

Since the forest fire case studied in this paper occurred in the Greater Khingan Mountains region of Heilongjiang Province, Wang Zhengfei’s forest fire spread model was adopted. This model is the result of Wang Zhengfei and colleagues’ extensive research on the characteristics of forest fire spread in the Greater Khingan Mountains region, and it has strong applicability in this area^14^. The calculation formula of the model is as follows:

1 $${v}_{0}=aT+bW+C$$

2 $${v}_{i}={v}_{0}{k}_{s}{k}_{w}{k}_{\varphi }={v}_{0}{k}_{s}{k}_{\varphi }{e}^{0.1782{v}_{w}}$$

The formula for extinguishing time relates to the rate of fire spread v_i_ at the point of ignition i, the time t_i_^k^ for the fire fighting vehicle to arrive at the point of ignition, and the rate of extinguishing v_f_^k^ at which the fire fighting vehicle extinguishes the point of ignition. The specific formula is shown below:

3 $${t}_{i}^{k}({t}_{i}+{t}_{i}^{k})\times {v}_{i}={t}_{i}\times {v}_{f}^{k}$$

The above formula can be deformed to obtain the fire extinguishing time calculation formula for the fire point as shown below:

4 $${t}_{i}=\frac{{t}_{i}^{k}{v}_{i}}{({v}_{f}^{k}-{v}_{i})}$$

In which the fire extinguishing speed of the fire engine must meet greater than the rate of spread of the fire at the point of ignition, in order to ensure that the fire is extinguished.

## Mathematical model

To establish an optimal model more effectively, the following assumptions are proposed:

1. Firefighting resources are limited, and the number of fire trucks is insufficient to cover all fire points.
2. All fire trucks have the same performance, and their firefighting speed is higher than the spread speed of the fire, ensuring they can extinguish all fire points.
3. In the case of a large-scale forest fire, each fire point occurs independently and does not affect each other.
4. The faster the fire spreads at a fire point, the more severe the fire, and the higher its rescue priority.
5. Fire trucks depart from the fire station and return to the station after completing all firefighting tasks.
6. The spread speed, number of fire points, and distances between them are known, and each fire point can only be serviced by one fire truck.
7. For each fire truck, the first rescue target point is assigned based on rescue priority. Assuming there are K fire trucks, the top K fire points in priority are evenly distributed to each fire truck as the first rescue target, while subsequent rescue targets for each fire truck do not need to be sorted by rescue priority.

Based on the above assumptions, to achieve the goal of minimizing the rescue time for fire trucks, the mixed-integer linear programming (MILP) model for the research problem can be established as follows:

5 $${\text{minT}}_{\text{max}}$$

6 $${\text{s}}.{\text{t}}.\;\mathop \sum \limits_{{k \in K}} \mathop \sum \limits_{{i \in V}} x_{{ij}}^{k} = 1,\forall i \ne j \in V^{\prime }$$

7 $$\sum_{j\in V}{x}_{0j}^{k}=1,\forall k\in K$$

8 $$\sum_{i\in V}{x}_{i0}^{k}=1,\forall k\in K$$

9 $$\sum_{i\in V}{x}_{ij}^{k}=\sum_{i\in V}{x}_{ji}^{k},\forall k\in K,\forall j\in {V}^{\prime}$$

10 $$\sum_{\text{i}\in \text{V}}\sum_{\text{j}\in \text{V}}\frac{{\text{d}}_{\text{ij}}}{{\text{v}}_{\text{k}}}{\text{x}}_{\text{ij}}^{\text{k}}\le {\text{T}}_{\text{k}},\forall \text{k}\in \text{K}$$

11 $$\sum_{\text{j}=1}^{|\text{K}|}\sum_{\text{k}\in \text{K}}{\text{x}}_{0\text{j}}^{\text{k}}=|\text{K}|$$

12 $${t}_{0}^{k}={T}_{0},\forall k\in K$$

13 $$t_{i} = \mathop \sum \limits_{{k \in K}} t_{i}^{k} \frac{{v_{i} }}{{\left( {v_{f}^{k} - v_{i} } \right)}},\forall \in V^{\prime }$$

14 $${t}_{i}^{k}-{t}_{j}^{k}-{t}_{j}-\frac{{d}_{ji}}{{V}_{k}}\ge M({x}_{ji}^{k}-1),\forall k\in K,\forall i\in {V}^{\prime},\forall j\in V$$

15 $${t}_{i}^{k}-{t}_{j}^{k}-{t}_{j}-\frac{{d}_{ji}}{{V}_{k}}\le M(1-{x}_{ji}^{k}),\forall k\in K,\forall i\in {V}^{\prime},\forall j\in V$$

16 $${C}_{max}\ge \sum_{k\in K}{t}_{i}^{k}+{t}_{i},\forall i\in {V}^{\prime}$$

17 $${t}_{i}^{k}\le M\sum_{j\in V}{x}_{ji}^{k},\forall k\in K,\forall i\in {V}^{\prime}$$

18 $$\mathop \sum \limits_{{j \in V}} x_{{ij}}^{k} \le 1,\forall k \in K,\forall i \in V^{\prime }$$

19 $$\mathop \sum \limits_{{j \in V}} x_{{ji}}^{k} \le 1,\forall k \in K,\forall i \in V^{\prime }$$

20 $${\text{t}}_{\text{i}}^{\text{k}}\ge 0,\forall \text{k}\in \text{K},\forall \text{i}\in \text{V}$$

21 $$t_{i} \ge 0,\forall i \in V^{\prime }$$

22 $${\text{x}}_{\text{ij}}^{\text{k}}\in \{\text{0,1}\},\forall k\in K,\forall i,j\in V$$

Among them, Eq. (5) represents the optimization objective, which is to minimize the overall firefighting time of the fire trucks. Equation (6) ensures that each fire point can only be serviced by one fire truck. Equations (7) and (8) ensure that fire trucks depart from the fire rescue base and return to the base after completing the firefighting. Equation (9) indicates that under the flow balance constraint, once a fire truck services a fire point, it must leave after extinguishing the fire. Equation (10) represents the travel time constraint for the fire trucks. Equation (11) indicates the priority dispatch of fire trucks to extinguish the top |K| fire points with higher rescue priority. Equation (12) represents the departure time of fire trucks from the rescue base. Equation (13) is the calculation formula for the firefighting time at fire points. Equations (14) and (15) calculate the travel time of fire trucks to the fire points. Equation (16) represents the total rescue time of fire trucks. Equation (17) describes that the arrival time of fire trucks at fire points is affected by whether the fire trucks arrive at those points. Equations (18) and (19) represent valid inequalities, indicating that each fire point can be serviced by fire trucks at most once. Equations (20), (21), and (22) specify the range of values for the decision variables.

## Algorithm design

**Fig. 1:**
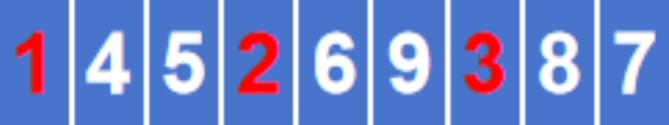
Overview of the Coding Design. Assume there are 9 fire points (N = 9) and 3 fire engines (k = 3). For the 9 fire points, an integer from 1 to 9 represents both the fire point identifier and the fire point rescue priority, with the rescue priority decreasing from 1 to 9. A higher rescue priority indicates a faster spreading fire. Each position in the sequence of integers represents information about a fire point or fire engine, resulting in a feasible solution.

To address the emergency response to a forest fire, the solution must be encoded, which is divided into two key parts: the number of fire trucks and the number of ignition points. A sequence of N-bit integers (N_1_, N_2_,…, N_n_) represents the genetic encoding, where the integers encompass information related to the fire points and fire engines. In this specific problem, it is assumed that there are 9 fire points (N = 9) and 3 fire engines (k = 3). For the 9 fire points, the integers from 1 to 9 not only represent the fire point identifiers but also reflect the rescue priority of the fire points, which decreases from 1 to 9. A higher rescue priority indicates a faster spreading fire. Each position in the integer sequence represents information about a fire point or fire engine, thus forming a feasible solution. The format of the encoding is shown below. As shown in Fig. 1.

For vehicle path planning for emergency rescue, this paper adopts the principle of partial rescue priority, taking the above code as an example. There are 3 fire engines, the number of fire points for 9, for 3 fire engines, the path to be planned for 3, each path in accordance with the principle of partial rescue priority, take the highest priority of the first 3 fire points as 3 fire engines rescue the first target, that is, each path from the fire station after the departure of the first rescue location, for the path of the subsequent fire point rescue target does not need to be in accordance with the priority of the rescue to sort. For the above coding, the 3 paths represented are the first fire engine: 1-4-5, the second fire engine: 2-6-9, and the third fire engine: 3-8-7. For the genes that are used as the first target of rescue in the coding, this paper refers to them as the rescue priority genes.

In this paper, a homogeneous strategy is introduced, i.e., a rounding function m = [n/k] is used to distribute the fire points to the fire and rescue vehicles in a homogeneous way. In other words, by dividing the number of fire points n by the number of firefighting vehicles k and rounding down the number of firefighting vehicles, a fair allocation of fire points is realized. From the perspective of gene coding, it can be regarded as assigning an even number of genes after each rescue priority gene, and each rescue priority gene and its assigned gene as a gene segment of an individual, and all the gene segments corresponding to all rescue priority genes are composed to obtain a complete individual. Through such a coding method, It can not only adapt to the needs of the research problem in this paper, but also effectively improve the rescue efficiency of the fire engine and reduce the maximum time required to extinguish the fire in the fire engine.

The algorithm in this paper takes a power function carrier approach to chaotic search based on the ordinary chaotic search to generate a genetic initial population. Let the initial generated individual be x_n_ (n = 0), which contains m codes, each code is regarded as a chaotic vector, then the individual x contains m chaotic vectors, x_k, n_(k = 1, 2, 3,……, m; n = 0). The chaotic search initialization enhances population diversity by mapping genetic codes into chaotic intervals using Eq. (23). This technique prevents premature convergence by ensuring a more comprehensive exploration of the solution space during the initial generation phase.

1. Since the coded numbers in the individual are in the form of positive integers and the range of values of x_k_, _n_ in the chaotic search is (0,1) and x_k_, _n_ cannot be equal to 0,0.25,0.5,0.75,1. Therefore, for the genetically coded values in the individual need to be mapped in a special way and then used. The range of values of x_k_, _n_ is called chaotic interval. Then the first step needs to map the gene coding values to chaotic intervals to get the chaotic mapping table, take x_5, 1_ individual (3,2,1,5,4) as an example, which contains the number of coding is 5, and the coding values take the value range [a, b] as [1, 5].
  The chaotic interval mapping for chaotic search is determined by dividing the chaotic interval *k* equally, as specified in Eq. (23).
  23 $${z}_{k}=\frac{{x}_{k,n}-a}{b-a}$$
2. The chaotic vectors in the individual are mapped to chaotic sequences through the mapping relation, and then the chaotic sequences are computed through Eq. (24) to find out the newly generated chaotic sequences after the iteration of the current position.
  24 $${x}_{k,n+1}=r\cdot {x}_{k,n}\cdot (1-{x}_{k,n}) (k=\text{0,1},2,... ...,m)$$
  Based on the power function carrier theory, the chaotic sequence x at the two ends of the interval is carrier processed to obtain the post-carrier chaotic sequence x^v^. The power function is a constant function of the segmented constant function, where is the segmented constant function.
  as in Eq. (25), where v is a segmented constant function.
  25 $$\text{v}=\left\{\begin{array}{c}0.5,asx\in (\text{0,0.15}]\\ 1.0,asx\in (\text{0.15,0.96}]\\ 6.29,asx\in (\text{0.96,1}]\end{array}\right.$$
3. Reflect the chaotic sequence into the positive integer form of the chaotic vector by judging the location of the chaotic interval in which the k chaotic sequences generated iteratively are located.
4. Repeat the above steps for the new individuals generated by chaotic search until the number of individuals generated by chaotic search reaches the maximum capacity of the initial population, and then the operation of generating the initial population based on chaotic search of power function carriers ends.

## Cross-variant operational

The specific steps of the crossover operation are as follows:

Individuals of the two parent generations are not F1{x_1,1_,x_1,2_,x_1,3_,…,x_1,9_} and F2{x_2,1,_x_2,2_,x_2,3_,…,x_2,9_},assuming that the number of genes coding in the parent individuals is 9 and the rescue priority genes are x_i,1_,x_i,4_,x_i,7_. Then follow the steps below to generate the offspring individuals C1 & C2:

STEP1: Extract the rescue genes x_i,1_, x_i,4_, x_i,7_ in the parent individual and put them into the offspring individual to get the infrastructure of the offspring individual.

STEP2: Extract the other genes except the rescue priority gene of the two parent individuals respectively, and arrange them closely in their original order to form two new individuals NF1{x_2,2_,x_2,3_,x_2,5_,x_2,6_,x_2,8_,x_2,9_},NF2{x_1,2_,x_1,3_,x_1,5_,x_1,6_,x_1,8_,x_1,9_},and then randomly select a gene x or x as the initial crossover position of the cyclic crossover from the new individuals.

STEP3: choose one of x_1,j_,x_2,j_ as the starting point of cyclic crossover, here assume that the selected crossover position is x_1,3_, the starting point of cyclic crossover is x_1,3_, then exchange x_1,3_ with x_2,3_, after exchange, go back to the parent NF1 to find the same coding position as the x_2,3_ gene and exchange, the cycle repeats, and ultimately find the coding position of the parent NF2 that is the same as the x_1,3_, so that the cyclic crossover step is over, and we get two new parent generations. NF1, NF2 crossover generated individuals NC1, NC2.

STEP4: Distribute the genes in the offspring NC1, NC2 to the offspring C1, C2 according to the original order and the number of genes assigned to the rescue priority genes in the parent generation F1, F2, and form the complete offspring C1, C2 with the rescue priority genes, and then the crossover operation is finished. Output the final offspring C1, C2.

**Fig. 2:**
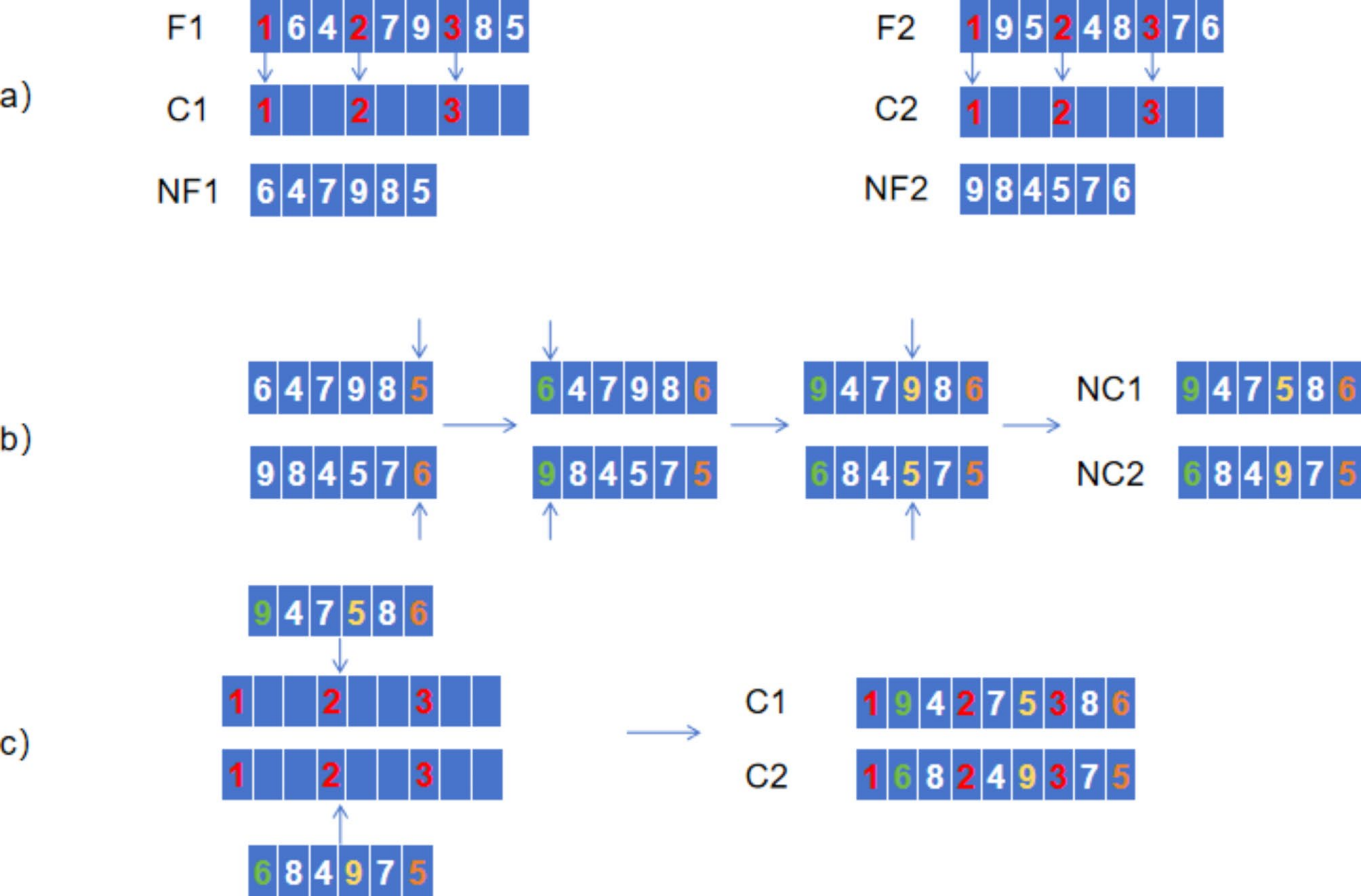
Overview of Improved Genetic Algorithm Crossover Operations. Firstly, the base structure of the offspring individual is obtained, then the crossover position is selected and the cyclic crossover operation is started, then in order to avoid the defects that exist in cyclic crossover that lead to the generation of offspring individuals that are the same as their parents, the offspring are generated and then processed for detection and finally the genes are assigned.

The process of crossover operation is demonstrated as shown in Fig. 2, where it is assumed that coding 1,2,3 are rescue priority genes. As shown in Fig. 2.

## Variant operation

**Fig. 3:**
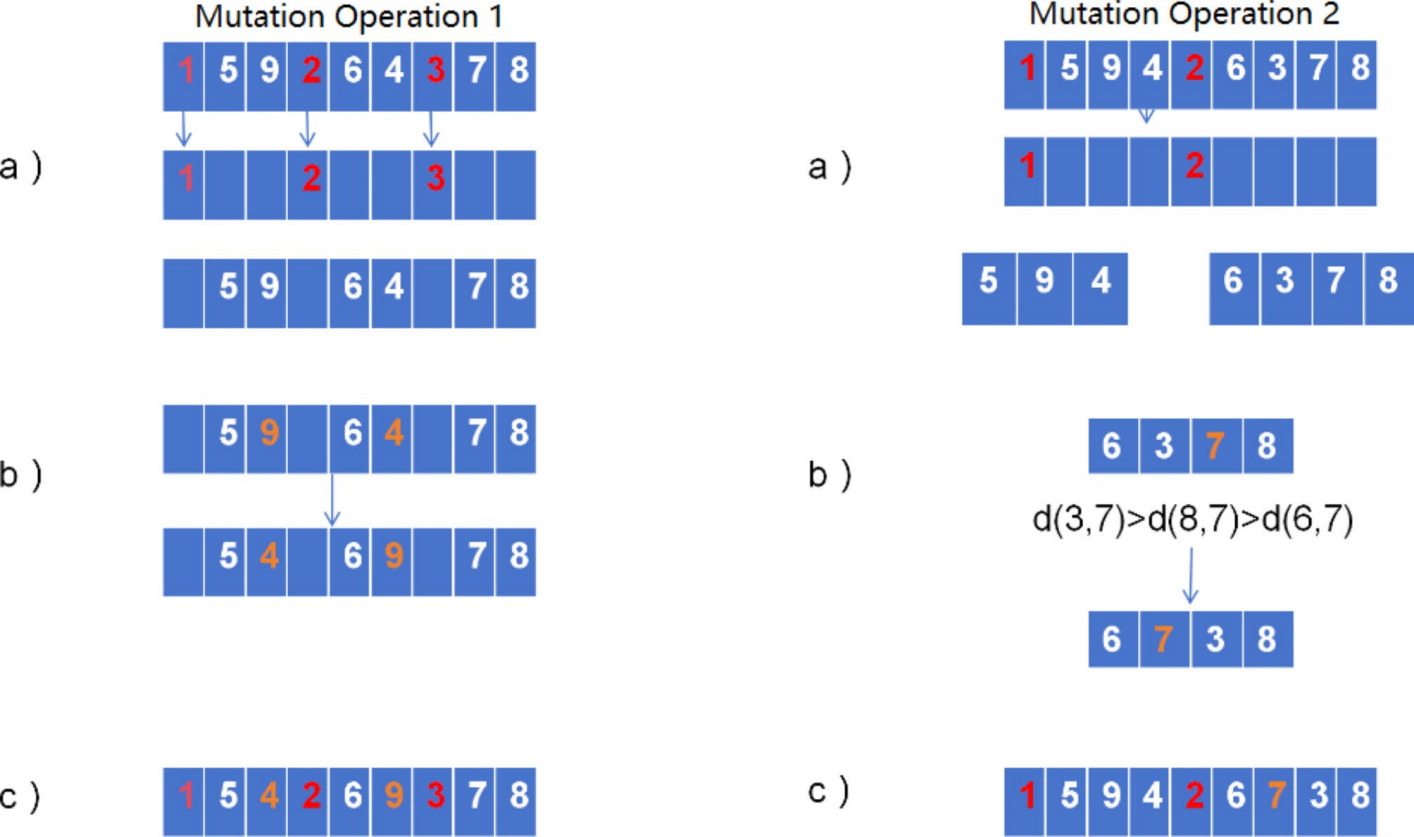
Overview of Improved Genetic Algorithm Mutation Operations. Mutation operation 1 mutation operator adopts two-point switched mutation, which targets the global and does not take into account the genes with subordinate properties in them, and finds the solution that is more different from the original individual so that there can be a certain probability of jumping out of the local optimum. Mutation operation 2 mutation operator adopts subregion shift operator, instead of arranging them closely as a new individual, it divides them into 3 gene segments according to the number of rescue priority genes, and randomly takes one of the 3 gene segments for mutation.

The traditional mutation operations in genetic algorithms have random single-point mutation and multi-point mutation, and with the increase of chromosome length, the range of randomly selected mutation positions in the mutation operation expands, which may lead to more ineffective searches, thus slowing down the algorithm’s iteration speed. In addition, the expansion of random mutation locations may also make it more difficult for the algorithm to achieve local optimization, which affects the performance of the algorithm. Due to the existence of special rescue priority genes in the research problem of this paper, the rescue priority genes cannot be changed throughout the process of the genetic algorithm. Based on the above problems and the characteristics of the research problem in this paper, this paper adopts two mutation operators in the mutation operation The specific operation steps of the two mutation operators are shown in Fig. 3.

The first mutation operator adopts two-point exchange mutation, the specific operation method is that the mutated individual extracts the genes other than the rescue priority gene, arranges them closely according to the original order to become a new individual, and randomly selects two mutation points x_m_ and x_n_ in the new individual, and then exchanges the position of x_m_ and x_n_ to complete the mutation.

The second mutation operator takes the shift operator of subregion, which operates as follows: assuming that the gene segment is {x_1_,x_2_,x_3_,x_4_} a gene x_2_ is randomly selected in the gene segment, comparing its relative distance d(x_1_,x_2_),d(x_3_,x_2_),d(x_4_,x_2_) with the other genes in the gene segment, and then moving the gene x_2_ after the gene with the smallest relative distance d, the mutation is completed, and the mutated individual is obtained by putting the mutated portion back to the original individual.

During the selection process with the two-population mechanism, the original population undergoes roulette selection after several iterations. Outstanding individuals from these iterations, referred to as elite individuals, are retained for further operations. After a certain number of iterations of the original population, In each selection operation, select the first N elite individuals in the population with higher fitness, add them to the elite population, the maximum capacity of the elite population is half of the original population, and when the population of the elite population reaches the maximum number of individuals, stop adding individuals, and the original population to form a two-population. Then synchronized cross mutation operation is performed on the two populations, and the two populations are consistent in the cross mutation operation. And there is a difference in the selection operation.

The original population was subjected to a roulette wheel selection method, and the probability Q_i_ of each individual being selected was calculated as shown below.

26 $$\text{P}({\text{X}}_{\text{i}})=\frac{\text{f}({\text{X}}_{\text{i}})}{\sum_{\text{j}=1}^{\text{M}}\text{f}({\text{X}}_{\text{j}})}$$

27 $${Q}_{i}=\sum_{j=1}^{i}P({X}_{j})$$

For the elite retention strategy in the elite population, the process is as follows:

Step1: Evaluate individual fitness: Evaluate the fitness of all individuals in the elite population and determine the fitness value of each individual.

Step2: Select elite individuals: Select the individuals with the highest fitness from the elite population as elite individuals.

Step3:Retain elite individuals: The selected elite individuals are directly copied to the next generation population, which can ensure that at least some of the individuals in the next generation population are excellent, thus preventing the algorithm from falling into a local optimal solution.

Step4: Fill the remaining population: In addition to the elite individuals, the remaining empty spaces in the population can be filled by cross mutation operation.

## Evaluation of adaptation

Since the optimization objective in this paper is to minimize the rescue time, the inverse of the objective function is used as the objective function for the calculation of the fitness.

28 $$fitness=\frac{1}{T}$$

### Case study

In this paper, a real fire case that occurred in July 2020 within the jurisdiction of Wuma Forestry Bureau and Yonganshan Forestry Bureau in Great Khingan is used for experimental simulation, and due to the lack of a suitable arithmetic example for the problem of emergency rescue vehicle dispatching for forest fires, a large-scale randomly generated fire point is used for experimental simulation as a simulation arithmetic example. In this chapter, the real fire case data are obtained from the local management bureau, and the large-scale simulation example is solved by randomly generating fire points 20,30,40 under the same example settings using IABC, GA, IGA algorithms, respectively, and the specific parameter settings are described in the following content.

The algorithms used and designed in this paper are implemented in python language, the operating system used for the experiment is Windows 10, CPU is 2.40 GHz and memory is 16 GB.

**Fig. 4:**
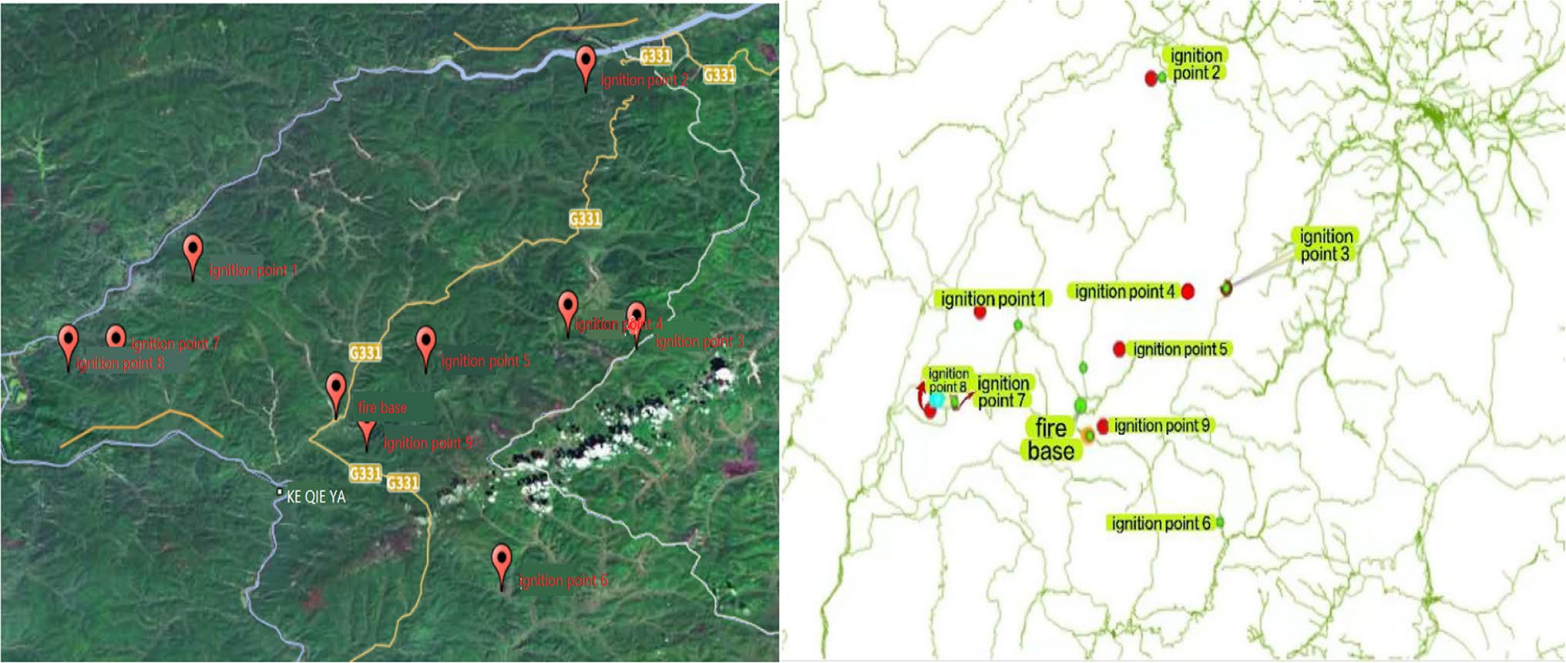
The image on the left is an image of the study area, and the image on the right is a road network map of the study area. There are a total of nine fires in this area.

The case studied in this paper is a number of fires that occurred in July 2020 in the area under the jurisdiction of Wuma Forestry Bureau and Yonganshan Forestry Bureau in Great Khingan, which can be regarded as a concurrent forest fire due to the fact that the fires occurred on the same day and the time interval between fires was relatively short. The forest fire is a typical lightning strike forest fire. It was a fire started by a natural summer lightning strike. This fire involves 9 ignition points, let N = 9, these 9 ignition points involved in the distribution of forest farms Ania Forest Farm, Jixinggou Forest Farm, Maoha Forest Farm 79 forest class, Maoha Forest Farm 96 forest class, Wuyuan Forest Farm, Yakruqi Forest Farm, Imuhe Forest Farm 34 forest class, Imuhe Forest Farm 42 forest class, and Uma Forest Farm. Fig. 4 is an image showing the distribution of fire points and the road network map of the study area.

The road network density in the northern primary forest area is low, and fire points are located far from the roads, making it impossible for fire trucks to directly reach the fire points using existing roads. To address this issue, this paper selects a position on the road surrounding the forest area as a connection point between the road and the forest area, as indicated by the green markers in the road network map above. The entire path from the fire station to the fire point is divided into two parts at the connection point. The first part starts from the fire station and ends at the connection point, while the second part starts from the connection point and ends at the fire point. The complete path from the fire station to the fire point is formed by merging these two parts. The planning of these two parts of the path is achieved by obtaining the road network data and DEM data of the study area, extracting the terrain slope and undulation information from the DEM data. Using ArcGIS, a shortest path analysis and a cost path analysis are conducted. The shortest path analysis determines the shortest path from the fire station to the connection point, while the cost path analysis identifies the path with the least obstruction from the connection point to the fire point.

## Analysis

### Shortest path analysis

**Fig. 5:**
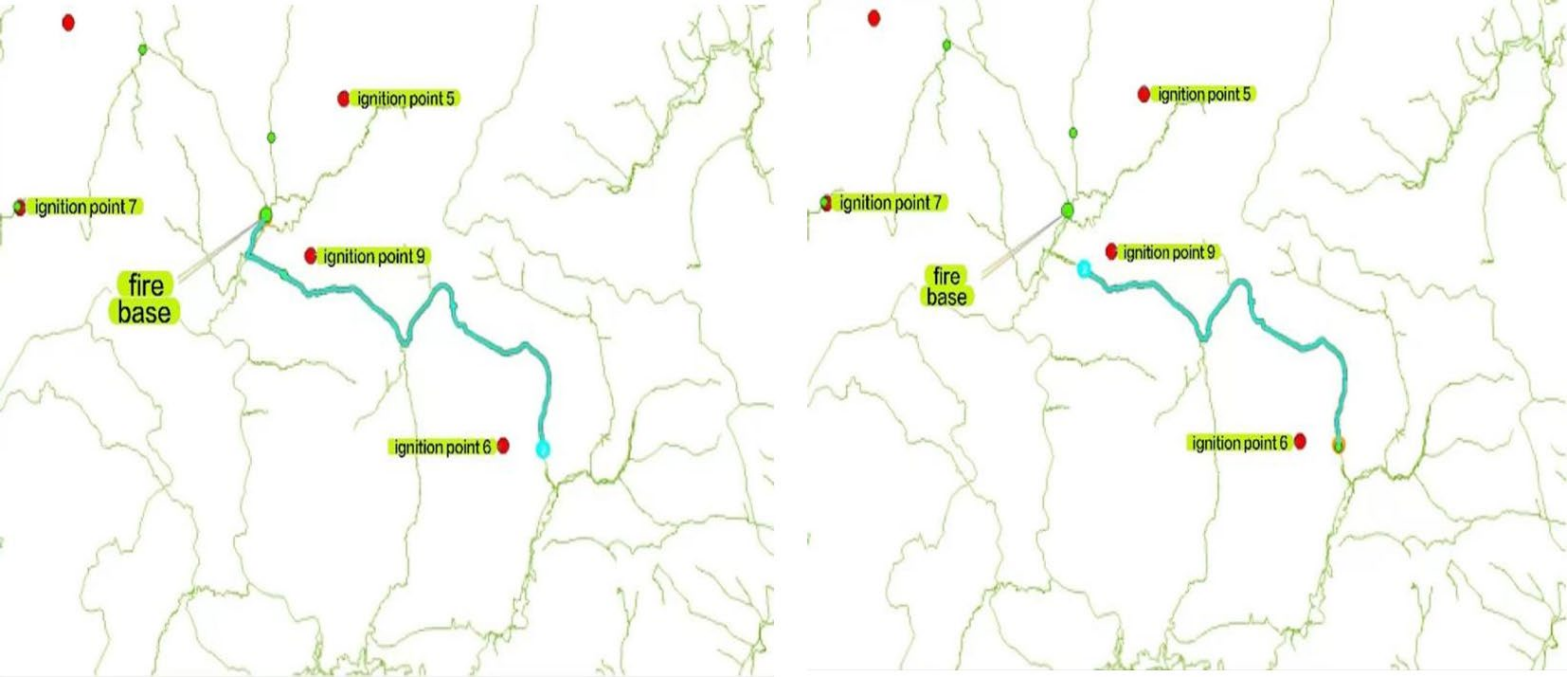
The above road network map shows the shortest path between the fire base and fire point 6 and the shortest path between fire points 6 and 9.

The shortest path analysis is conducted using the Network Analyst tool in ArcGIS to determine the shortest paths between fire stations and connection points, as well as between connection points and various fire points. Taking fire point 6 and fire point 9 as examples, the following two figures illustrate the planned routes after performing the shortest path analysis. As shown in Fig. 5.

### Cost path analysis

**Fig. 6:**
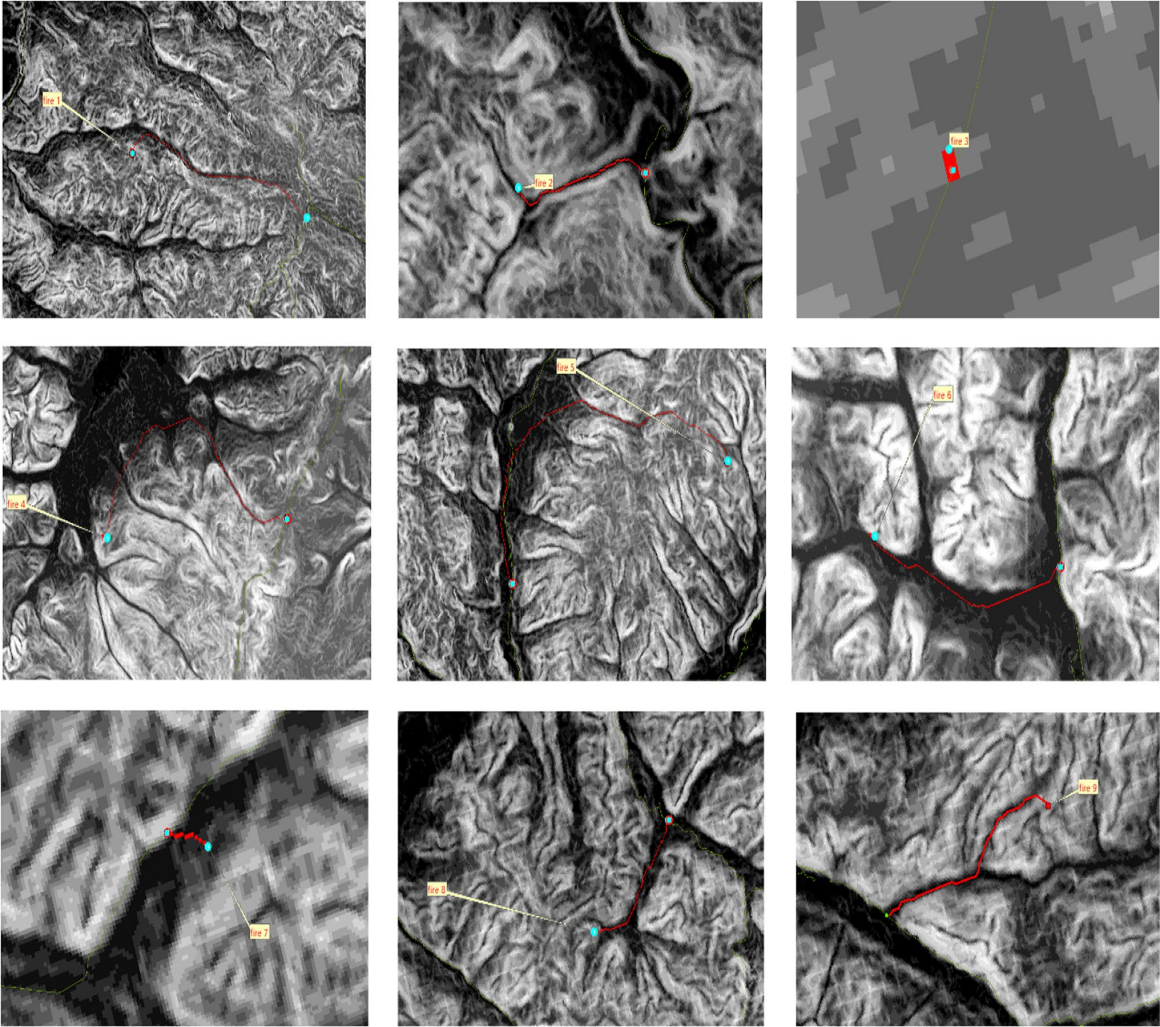
The above image shows the cost path analysis for fire points 1–9 using the Spatial Analyst tool.

The cost path analysis between fire points and connecting points was conducted using ArcGIS. The weighting factors applied were slope and undulation, with equal weights assigned in the following formula: “Slope” * 0.5 + “Undulation” * 0.5. The final path is generated by using the end point data, the cost distance data, and the linkback data, and the “cost path” of “Distance analysis” in the tool of “Spatial Analyst”. Distance Analysis” in the Spatial Analyst tool to generate the final path. The generated path is shown in the figure below. As shown in Fig. 6.

The fire spread rate of the ignition point was calculated based on Wang Zhengfei’s forest fire spread model as shown in the table below. As shown in Table 2, 3 and 4 below.

**Table 2:** The fire spread rate of the ignition point was calculated based on Wang Zhengfei’s forest fire spread model as shown in the following table.

${V}_{i}$	1	2	3	4	5	6	7	8	9
Rate of spread	1.1	3.2	0.8	3.0	2.2	7.8	0.9	1.6	4.9

**Table 3:** According to the speed of fire spread will be ranked, after the ranking of the fire point 1 for the rescue of the highest priority fire point, the rest of the fire point rescue priority decreasing step by step.

${V}_{i}$	1	2	3	4	5	6	7	8	9
Rate of spread	7.8	4.9	3.2	3.0	2.2	1.6	1.1	0.9	0.8

**Table 4:** 1–9 Distance between ignition points by priority of rescue.

*V*_*i*_	0	1	2	3	4	5	6	7	8	9
0	0	79	17	468	541	27	79	70	41	540
1	79	0	73	453	540	107	159	150	121	524
2	17	73	0	461	550	44	96	87	58	533
3	468	453	461	0	126	496	548	535	506	109
4	541	540	550	126	0	584	636	627	598	17
5	27	107	44	496	584	0	109	96	68	567
6	79	159	96	548	636	109	0	11	54	619
7	70	150	87	535	627	96	11	0	54	610
8	41	121	58	506	598	68	54	54	0	581
*V*_*i*_	0	1	2	3	4	5	6	7	8	9
9	540	524	533	109	17	567	619	610	581	0

### Simulation results analysis

The cases in this chapter set up different scenarios by varying the speed of extinguishing the fire and the speed of traveling of the rescue vehicle. The number of rescue vehicles in all scenarios is 3, and the setup scenarios are shown below:

Scenario 1: The fire extinguishing speed is 8 min/m and the rescue vehicle traveling speed is 54 km/h.

Scenario 2: The extinguishing speed is 8 min/m and the speed of the rescue vehicle is 74 km/h.

Scenario 3: The fire is extinguished at 10 min/m and the rescue vehicle is traveling at 54 km/h.

Scenario 4: The fire is extinguished at 10 min/m and the rescue vehicle is traveling at 54 km/h.

Scenario 5: The fire is extinguished at 12 min/m and the rescue vehicle is traveling at 54 km/h.

Scenario 6: The fire is extinguished at 12 min/m and the rescue vehicle travels at 54 km/h.

Based on the small size of the simulation case, the number of iterations is set too large to reflect the effectiveness of the algorithm, so the number of iterations of the algorithm used in all scenarios is set to 10, in order to compare the advantages and disadvantages of the solution obtained in the shortest time in the small-scale case, and better reflect the performance of the algorithm.

After solving the six scenarios set up by the experimental simulation using the IABC, GA, IGA algorithms respectively, the calculated results are shown in the table below, where Best is the optimal value of the objective function obtained after the algorithm has been run for 100 times, Avg is the average value of the objective function, D is the distance traveled in the whole rescue trip, and CPU is the average running time of the program. See Table 5 and 6 below.

**Table 5:** The following table shows the computational results obtained by solving the nine scenarios set up by the experimental simulation using IABC, GA, IGA algorithms respectively.

Sight	$v_f^k$	$v^{k}$	Avg	D	Best	Cpu/ms
IABC						
1	10	64	25.6	2596	25.6	10
2	10	74	22.1	2596	22.1	9.6
3	12	64	23.7	2596	23.7	8.4
4	12	74	20.5	2596	20.5	8.1
5	14	64	22.5	2596	22.5	8.6
6	14	74	19.5	2596	19.5	8.8
GA						
1	10	64	27.2	2587	27.25	12
2	10	74	22.3	2845	22.3	11.6
3	12	64	24.8	2567	24.8	10.5
4	12	74	21.4	2583	21.45	10.6
5	14	64	23.4	2567	23.4	9.9
6	14	74	20.2	2567	20.2	9.4
IGA						
1	10	64	25.5	2580	25.5	9.8
2	10	74	22	2580	22	9.4
3	12	64	23.6	2580	23.6	8.4
4	12	74	20.4	2580	20.4	7.9
5	14	64	22.4	2580	22.4	8.4
6	14	74	19.4	2580	19.4	8.2

*Note*: Best is the optimal value of the objective function obtained after the algorithm has been run 100 times, Avg is the average value of the objective function, D is the distance traveled for the entire rescue trip, and CPU is the average running time of the program.

**Table 6:** Fire truck scheduling scheme for 9 scenarios using IABC, GA, IGA algorithm.

Sight	Fire engine A1	Fire engine A2	Fire engine A3
1	0-1-5-6-0	0-2-4-9-0	0-3-7-8-0
2	0-1-5-6-0	0-2-4-9-0	0-3-7-8-0
3	0-1-5-6-0	0-2-4-9-0	0-3-7-8-0
4	0-1-5-6-0	0-2-4-9-0	0-3-7-8-0
5	0-1-5-6-0	0-2-4-9-0	0-3-7-8-0
6	0-1-5-6-0	0-2-4-9-0	0-3-7-8-0

Sight	Fire engine B1	Fire engine B2	Fire engine B3
1	0-1-5-7-0	0-2-4-9-0	0-3-6-8-0
2	0-1-5-6-0	0-2-7-4-0	0-3-9-8-0
3	0-1-7-5-0	0-2-9-4-0	0-3-8-6-0
4	0-1-5-7-0	0-2-4-9-0	0-3-8-6-0
5	0-1-5-7-0	0-2-9-4-0	0-3-8-6-0
6	0-1-7-5-0	0-2-9-4-0	0-3-8-6-0

Sight	Fire engine C1	Fire engine C2	Fire engine C3
1	0-1-5-6-0	0-2-9-4-0	0-3-8-7-0
2	0-1-5-6-0	0-2-9-4-0	0-3-8-7-0
3	0-1-5-6-0	0-2-9-4-0	0-3-8-7-0
4	0-1-5-6-0	0-2-9-4-0	0-3-8-7-0
5	0-1-5-6-0	0-2-9-4-0	0-3-8-7-0
6	0-1-5-6-0	0-2-9-4-0	0-3-8-7-0

Sequences represent truck routes, e.g., '0-1-5-6-0' means starting from the station (0), visiting fire points 1, 5, and 6, then returning to the station.

*Note*: Where A1, A2, A3 are IABC algorithm solved to obtain the fire truck scheduling scheme, B1, B2, B3 are GA algorithm solved scheme, C1, C2, C3 are IGA algorithm solved scheme.

From the table, it can be seen that in the six scenarios set up, the optimal and average results calculated by the IGA algorithm are improved compared with the ordinary GA algorithm, and the Best value is reduced by about 4%-8%. The experimental results indicate that the IGA algorithm produces higher-quality solutions compared to the conventional GA algorithm, significantly reducing rescue times in emergency operations. While the improvement over the IABC algorithm in Best values is marginal, this can be attributed to the small scale of the test cases, and the adoption of the principle of partial rescue priority, without restricting the rescue order of other genes in addition to the rescue priority genes, has Expanding the scope of the solution enables the IGA algorithm to avoid falling into a local optimal situation. There is also a reduction in the average program running time of the IGA algorithm, which shows that the IGA algorithm is able to find high quality solutions quickly.

Due to the small size of the fire in the above real cases, the algorithm’s performance requirements are not high, and it is not possible to compare the algorithm’s performance. In order to better validate the algorithm’s performance, the simulation cases with 20,30, and 40 fires are randomly generated, and the IABC, GA, and IGA algorithms are used under the same parameter settings as follows: the fire spread speed is randomly generated within [1,7] m/min, and the distance between each fire point is randomly generated in [50,100] km.

The detailed scenario settings and solution results of the large-scale experimental simulation are shown in the table below, and the number of vehicles, K, is added as an additional parameter variable due to the large scale of the number of fire points compared to that in the previous experimental simulation. As shown in Table 7 below.

**Table 7:** The results of large-scale experimental simulations of the three algorithms are as follows.

Formula	V′	K	${v}_{f}^{k}$	${v}_{k}$	Avg	Best	Avg	Best	Avg	Best
IABC							GA		IGA	
1	20	5	12	54	19.7	19.5	20.9	20.7	18.9	18.7
2	20	5	10	64	26	25.7	27.1	26.4	25.1	24.6
3	20	5	14	64	13.2	13.1	13.6	13.5	12.6	12.5
4	30	6	12	54	42.1	41.6	43.9	43.1	40.3	39.6
5	30	6	10	64	68.6	67.2	73.9	73.1	65.9	65.2
6	30	6	14	64	23.9	23.7	25.5	25.3	23.2	22.6
7	40	8	12	54	30.8	29.9	32.5	31.4	28.8	28.5
8	40	8	10	64	40.8	40.7	43.5	42.6	39.8	39.6
9	40	8	14	64	18.6	18.5	20.1	19.7	18.2	18

*Note*: K is the number of fire engine vehicles, Best is the optimal objective function value obtained in 50 runs of the algorithm, Avg is the average objective function value obtained in 50 runs of the algorithm.

From the calculation results in the table, it can be found that the IGA algorithm in this paper in the calculation of the Avg value and the Best value is better than the IABC algorithm and the GA algorithm, which is not much difference between the Avg value and the Best value obtained by the IABC algorithm. The difference between the Avg and Best values of the IGA algorithm and the IABC algorithm is not significant. The numerical reduction of the total rescue time of the Best values of the IGA algorithm compared to the Best values of the IABC algorithm is up to 5%, with an average of about 3.5%, and the maximum reduction of the Best values compared to the GA algorithm is 10.9%, and the reduction of the Avg values is 8.8%. In all the large-scale example settings, the IGA algorithm shows an improvement in the results obtained by the algorithm compared to the IABC algorithm and the ordinary GA algorithm, which indicates that the IGA algorithm in this paper performs well in terms of computational results. In the running time of the program, the IGA algorithm performs better, which shows that the IGA algorithm can get the scheduling plan more quickly in the unexpected situation of forest fires, and reduce the damage caused by forest fires.

**Fig. 7:**
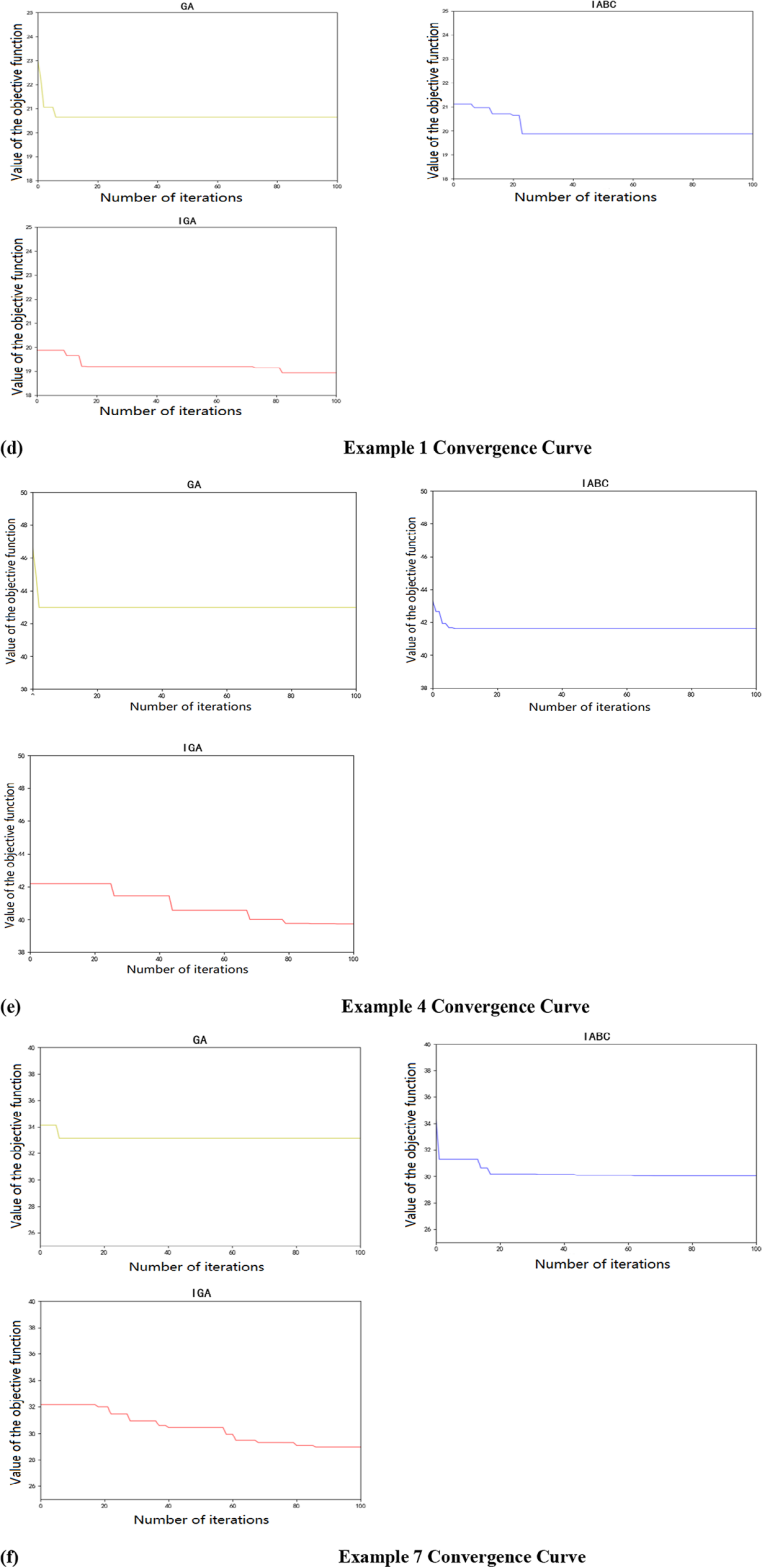
The above pictures show the convergence curves for arithmetic example 1, arithmetic example 4, and arithmetic example 7, accordingly. The above convergence curves show that the IGA algorithm gives a better solution compared to the other two algorithms at the beginning of the iterative process. Figure 7 demonstrates that the IGA algorithm outperforms GA and IABC by achieving faster convergence to optimal solutions, highlighting its efficiency in avoiding local optima and delivering higher-quality scheduling results.

The convergence curves after solving each case with GA, IABC and IGA algorithms are compared with the three cases in the large-scale case where the scenario is set to 54 km/h and the fire extinguishing speed is 12 m/min, and the convergence curves are shown in the figure below. As shown in Fig. 7.

The aforementioned discussion demonstrates that employing a power-function carrier-based chaotic search for generating initial solutions enhances the diversity and traversal capabilities of the initial population. This method increases the likelihood of obtaining high-quality solutions during the initial population generation phase. At the onset of the iterative process, all three algorithms exhibit rapid convergence, achieving relatively stable solutions after a certain number of iterations. The convergence diagrams indicate that the standard Genetic Algorithm (GA) tends to fall into local optima, making it challenging to search for better solutions. In contrast, the Improved Genetic Algorithm (IGA), which incorporates enhancements over the standard GA, effectively mitigates the risk of local optima entrapment. A comparative analysis of the three algorithms reveals that the IGA outperforms the other two in terms of the final results obtained from the convergence process across three experimental groups. The convergence graphs of the three algorithms further corroborate the superiority and effectiveness of the IGA in addressing the problem of scheduling vehicles for emergency rescue in forest fire scenarios.

## Conclude

In response to the unique challenges of road conditions and vehicle scheduling for forest fire rescue in the northern primary forest area, this paper establishes a vehicle scheduling model aimed at minimizing rescue time. Considering the actual road conditions in the northern Great Khingan primary forest area, characterized by a low highway network density and fire points located far from the roads, fire trucks cannot directly reach the fire points via the road network. To address this issue, this study downloads DEM data of the research area, extracting slope and undulation information as weight factors. Before solving the problem, the path with minimal obstruction is planned and then combined with the roads in the network. This integrated path is introduced into the problem model, and the traditional vehicle scheduling problem-solving methods are used, reducing the differences between forest fire rescue vehicle scheduling and traditional vehicle scheduling problems. This ensures the authenticity and reliability of the research results, providing a new approach to the emergency vehicle scheduling problem for forest fire rescue, thereby enhancing the practical significance of the study. A chaotic search method based on a power function carrier is introduced to generate the initial population, and the Improved Genetic Algorithm (IGA) is designed. Experimental simulations were conducted on real fire cases and randomly generated large-scale instances. By comparing the results of different algorithms, the feasibility and effectiveness of the IGA algorithm were verified. Future studies should explore the dynamic impact of climate change on fire frequency and intensity, as well as the integration of real-time environmental data into vehicle scheduling models.. The IGA algorithm efficiently generates high-quality solutions for emergency vehicle scheduling in forest fire rescue scenarios, enabling rapid response by rescue teams in sudden fire incidents. Using the feasible solutions derived from the algorithm, rescue vehicles can be promptly dispatched to mitigate the damage caused by forest fires, significantly enhancing emergency rescue capabilities in sudden forest fire scenarios.

## Data Availability

The datasets used and/or analysed during the current study available from the corresponding author on reasonable request.
